# Dietary Inflammatory Index and Odds of Colorectal Cancer and Colorectal Adenomatous Polyps in a Case-Control Study from Iran

**DOI:** 10.3390/nu11061213

**Published:** 2019-05-28

**Authors:** Pegah Rafiee, Nitin Shivappa, James R. Hébert, Saeede Jaafari Nasab, Alireza Bahrami, Azita Hekmatdoost, Bahram Rashidkhani, Amir Sadeghi, Mohammad Houshyari, Ehsan Hejazi

**Affiliations:** 1 Department of Clinical Nutrition and Dietetics, Faculty of Nutrition Sciences and Food Technology, National Nutrition and Food Technology, Research Institute, Shahid Beheshti University of Medical Sciences, Tehran 11369, Iran; pegahrafiee18@gmail.com (P.R.); saeejafari@gmail.com (S.J.N.); alirezabahrami38@yahoo.com (A.B.); a_hekmat2000@yahoo.com (A.H.); 2 Student Research Committee, (Department and Faculty of Nutrition Sciences and Food Technology), Shahid Beheshti University of Medical Sciences, Tehran 11369, Iran; 3 Cancer Prevention and Control Program, University of South Carolina, Columbia, SC 29208, USA; shivappa@email.sc.edu (N.S.); jhebert@sc.edu (J.R.H.); 4 Department of Epidemiology and Biostatistics, Arnold School of Public Health, University of South Carolina, Columbia, SC 29208, USA; 5 Connecting Health Innovations LLC, Columbia, SC 29201, USA; 6 Community Nutrition Department, Faculty of Nutrition Sciences and Food Technology, National Nutrition and Food Technology Research Institute (WHO Collaborating Center), Shahid Beheshti University of Medical Sciences, Tehran 11369, Iran; rashidkhani@yahoo.com; 7 Gastroenterology and Liver Diseases Research Center, Research Institute for Gastroenterology and Liver Diseases, Shahid Beheshti University of Medical Sciences, Tehran 11369, Iran; amirsadeghimd@yahoo.com; 8 Radio-Oncology Department, Shohadae Tajrish Hospital, Shahi Beheshti University of Medical Sciences, Tehran 11369, Iran; dr.mhoushyari@gmail.com

**Keywords:** colorectal cancer, colorectal adenomatous polyps, dietary inflammatory index, diet, inflammation

## Abstract

Background: Chronic inflammation is implicated in the development of colorectal cancer (CRC) and its precursor; colorectal adenomatous polyps (CAP). Some dietary factors are important triggers for systemic inflammation. Therefore, the present study aimed to investigate the association between the dietary inflammatory index (DII^®^) and the risk of CRC and CAP in an Iranian case-control study. Methods: 134 newly diagnosed CRC patients, 130 newly diagnosed CAP patients, and 240 hospitalized controls were recruited using convenience sampling. Energy-adjusted DII (E-DII) scores were computed based on dietary intake assessed using a reproducible and valid 148-item food frequency questionnaire. Logistic regression models were used to estimate odds ratios (ORs) and 95% confidence intervals (CI) after adjusting for confounders. Results: The E-DII score ranged between −4.23 (the most anti-inflammatory score) to +3.89 (the most pro-inflammatory score). The multivariable-adjusted ORs for participants in the 3rd tertile compared to the 1st tertile was 5.08 (95%CI: 2.70–9.56; P-trend < 0.0001) for CRC and 2.33 (95% CI: 1.30–4.02; P-trend = 0.005) for CAP. Conclusions: Our findings suggest that more pro-inflammatory diets, indicated by higher E-DII scores, might increase the risk of both CRC and CAP. Future steps should include testing these associations in a prospective setting in Iran.

## 1. Introduction

As per the GLOBOCAN [i.e., International Agency for Research on Cancer (IARC)] 2018 data, it is estimated that 1.8 million cases of colorectal cancer were diagnosed in 2018. Colorectal cancer (CRC) is the 3rd most common and the 4th most deadly cancer worldwide [1]. CRC is the 3rd and 4th most common cancer in Iranian females and males, respectively [2,3]. CRC is a multi-factorial chronic disease. Several modifiable factors (such as diet, cigarette smoking, physical inactivity, obesity and use of certain medications.) and non-modifiable factors (including age, gender, race, familial adenomatous polyposis, hereditary nonpolyposis colorectal cancer) can affect the risk of this cancer [4,5,6,7].

Inflammation usually occurs as part of the normal response to tissue insult or injury [8]. A significant body of evidence suggests that chronic inflammation and associated conditions such as inflammatory bowel disease play a central role in CRC and colorectal adenomatous polyps (CAP) pathogenesis by stimulating cell proliferation, angiogenesis and DNA damage [9,10,11]. Several mechanisms including increasing insulin resistance [12], and activation of the COX-2 pathway could provide ways to express diet-related inflammation in CRC and CAP [13]. Diet is a strong predictor of both inflammation and CRC [14,15,16,17,18]. Consumption of fruit, vegetables, fiber and moderate alcohol intake can decrease inflammation, whereas high consumption of red meat, processed meat and fat increase inflammation [19,20,21,22].

The dietary inflammatory index (DII^®^) is a literature-derived, population-based index that was developed to predict the inflammatory potential of diet [23,24]. The DII has been validated with various inflammatory markers, including C-reactive protein [17,24], interleukin-6 [25], and homocysteine [25]. The relationship between the DII and CRC has been established previously [10,11,19,26]], but only a few studies have examined the association between the DII and CRC in Middle Eastern countries [26,27]. It is well known that CAP are known precursors of CRC [28], Despite this, few studies have investigated the relationship between dietary factors and CAP [29,30,31]. To our knowledge, two previous studies in the US have examined the association between energy-adjusted DII (E-DII) and colorectal adenomas, but results were inconsistent [32,33]. Furthermore, no previous study has assessed the relationship between energy-adjusted DII (E-DII) and the risk of CAP. Thus, the aim of this study is to examine the relationship between E-DII and the risk of CRC and CAP in a case-control study in Iran.

## 2. Materials and Methods

### 2.1. Participants

This hospital-based case-control study was carried out in three referral hospitals in Tehran (capital of Iran). CAP cases were asymptomatic individuals, age 39–70 years, referred by their physicians for routine screening, rectal bleeding or having positive routine fecal occult blood test. A total of 134 patients undergoing colonoscopy and found to have pathologically confirmed CAP were recruited. CRC cases were patients undergoing colonoscopy and found to have a pathologically confirmed malignancy. They also were 30 to 79 years of age, diagnosed in the 3 months prior to the interview, and had no previous diagnosis of any other type of cancer or CAP (n = 139). Controls were enrolled randomly from among patients admitted to the same hospitals, at the same time as cases. Controls also were 30 to 70 years of age, with no diagnosis of neoplastic condition and were not on a special diet (n = 268). Controls were frequency matched on age (±10 years) and sex with cases. Of enrolled cases and controls, 28 controls and 5 CRC and 4 CAP cases were excluded because of incomplete food frequency questionnaires or extreme energy intake estimates that reflected careless completion of the dietary questionnaire (below or above the mean ±3 standard deviations for loge-transformed calories, see Supplementary Figure S1).

### 2.2. Inclusion and Exclusion Criteria

Inclusion criteria included the following: 1—Having no special diet that could influence the patient’s weight; 2—Absence of malignancy (any for the control or CAP group, prior cancer for the CRC group); 3—To be in age range of 20 to 70 years; 4—Willingness to participate in the study. Exclusion criteria included the following: 1—Pregnancy or lactation 2—Past medical history of malignancy or CAP.

### 2.3. Assessment of Dietary Intake

In this study, dietary intakes of participants over the past year were evaluated using a validated semi-quantitative food frequency questionnaire (FFQ) consisting of 148 food items [34]. Participants were provided with response categories for their frequency of consumption of each food item (daily, weekly, monthly and yearly) based on a standard portion size for each food item and then each participant’s reported intake was converted to weight equivalents (i.e., g, mg, ug) per day. The DII score was calculated using the method previously reported by Shivappa et al. [24]. Briefly, the scoring algorithm was based on extensive review of the literature published from 1950 through 2010, which focused on the effect of diet on six inflammatory biomarkers (IL-1β, IL-4, IL-6, IL-10, TNF-α, and CRP). A total of 45 food parameters, including macronutrients and micronutrients, were identified through the search and scored according to whether they increased (+1), decreased (−1), or had no effect (0) on these inflammatory biomarkers. These scores were weighted based on study design and were called inflammatory effect scores. To avoid the arbitrariness resulting from simply using raw consumption amounts, intakes of foods and nutrition were standardized to a representative range of dietary intakes based on actual human consumption in 11 populations living in different countries across the world that provided an estimate of a mean and standard deviation for each parameter.

**Figure 1:**
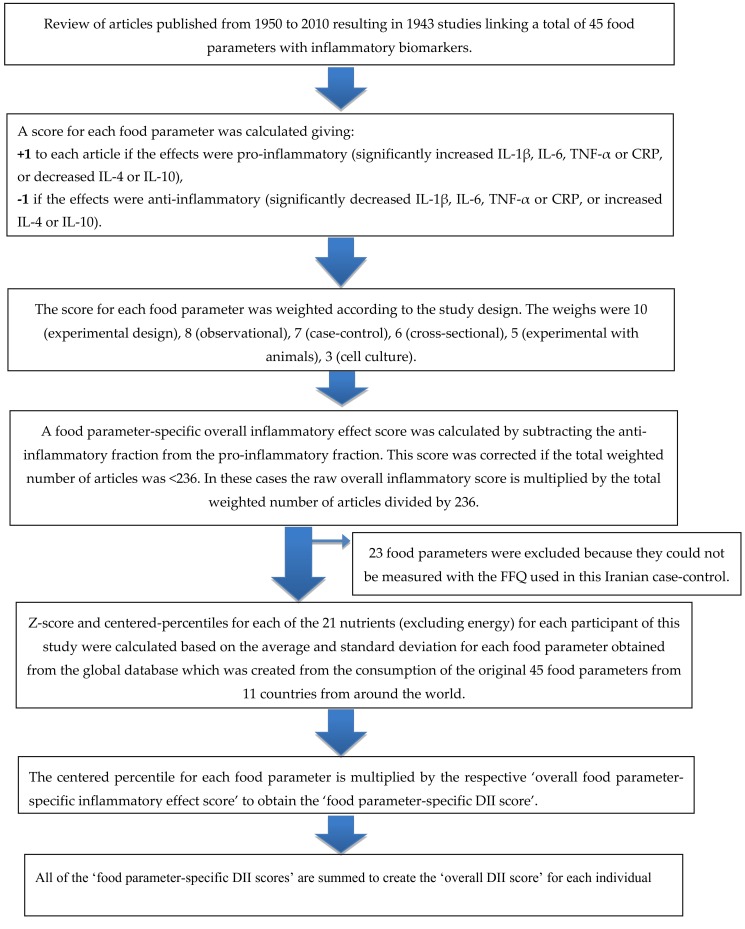
Sequence of steps in creating the energy-adjusted dietary inflammatory index (E-DII) scores in the Iranian colorectal cancer case-control study.

To compute the energy adjusted DII (E-DII) scores we converted all reported intakes of the food parameters to an amount per 1000 kcal of energy intake. This required using a version of the global comparative database in which all parameters are expressed per 1000 kcal of energy [35]. These values were then converted to a proportion (with values from 0 to 1). Each proportion was doubled, and then 1 was subtracted to achieve a symmetrical distribution around a mean of ≈0. For each individual food parameter, this score was then multiplied by the respective food parameter effect score, derived from the literature review, in order to obtain a food parameter-specific DII score. All of the food parameter-specific DII scores were then summed to create the overall DII score for every participant in the study [36], DII = b1*n1+b2*n2...........b21*n21, where b refers to the literature-derived inflammatory effects score for each of the evaluable food parameters and n refers to the food parameter-specific centered percentiles, which were derived from the dietary data. A description of validation work, including both dietary recalls and a structured questionnaire similar to an FFQ, also is available [37]. The methodology is depicted in Figure 1.

In a final step before analysis, tertiles of E-DII were created based on the baseline E-DII scores. Tertile 1 represents diets with the most anti-inflammatory potential. Tertile 2 is intermediate and tertile 3 represents the most pro-inflammatory diet. To calculate the E-DII scores in this study data were available for a total of 21 food parameters; i.e., all nutrients, including (carbohydrate, protein, total fat, fiber, cholesterol, saturated fatty acid, monounsaturated fatty acid, polyunsaturated fatty acid, vitamin A, vitamin B1, vitamin B2, vitamin B6, vitamin B12, vitamin C, vitamin D, vitamin E, folic acid, iron, magnesium, zinc and selenium. Previously, the E-DII has been validated with several inflammatory markers in various populations [25,35,38], including in Iran [39,40,41].

### 2.4. Assessment of Other Variables

All participants were interviewed to obtain information including socio-demographic characteristics, family history of cancer, including CRC, smoking habit, past medical history (comorbidities, medications and vitamin/mineral supplements intake) and usual cooking techniques. Each participant’s standing height, without shoes, was measured with a sensitivity of 0.1 cm. Weight was measured by a digital scale with 100 g sensitivity. All participants were asked to complete a physical activity questionnaire and rate their daily activities such as walking, exercise, sleep, watching television, housework and job-related tasks. Total activity was reported for 1 day, and the metabolic equivalent of tasks (METs) were calculated based on these reports.

### 2.5. Statistical Analysis

The Kolmogorov–Smirnoff test was used to evaluate whether or not the distributions of the variables were normal. Mean values for cases and controls were compared using the Student’s *t*-test and the means of more than two groups values were assessed using analysis of variance (ANOVA) for normally distributed variables. Non-parametric statistics, including the Mann–Whitney U test or Kruskal–Wallis test, were used for variables that were not normally distributed. The chi-square test was used for comparing distribution of categorical variables. Binary logistic regression was used to estimate odds ratios (ORs) and 95% confidence intervals (CIs) adjusted for multiple covariates in different models. E-DII scores were analyzed as both continuous and as quartiles. Statistical tests were performed using SPSS^®^ software (SPSS Inc., Chicago, IL). Odds ratio (OR) and 95% confidence interval (CI) were reported, and *p*-values <0.05 were considered statistically significant.

### 2.6. Ethical Approval

The protocol of the present study was approved by the Ethics Committee of Shahid Beheshti University of Medical Sciences with the ethic code of IR.SBMU.NNFTRI.REC.1397.041.

## 3. Results

The E-DII scores in this study ranged from −4.23 (the most anti-inflammatory score) to +3.89 (the most pro-inflammatory score). The socio-demographic and lifestyle characteristics of the subjects are presented in Table 1. Cases with polyps were significantly more likely to have comorbidities such as diabetes (*p* = 0.03), hypertension (*p* = 0.01) and coronary heart disease (*p* = 0.008)), less physical activity (*p* = 0.003), consume fewer steam-cooked foods (*p* = 0.006), have lower salt intake (*p* = 0.002) and higher daily intake of calcium supplements (*p* = 0.01) compared to controls. However, there were no significant differences in other variables such as body mass index (BMI), smoking, family history of cancer in first degree relatives, family history of colorectal cancer in first degree relatives, monthly intake of Vitamin D supplements and energy intake between CAP patients and controls. Compared with controls, patients with CRC were significantly more likely to have comorbidities such as diabetes (*p* = 0.001) and coronary heart disease (*p* = 0.009). CRC patients also reported more frequent family history of cancer in first-degree relatives (*p* = 0.001) and higher intake of salt (*p* = 0.002); however, they consumed significantly fewer fried foods (*p* = 0.001), grilled foods (*p* = 0.02) and foods cooked using multiple (i.e., combined) techniques (*p* = 0.001). There were no significant differences between CRC patients and controls. Cases (both those with CRC and those with CAP) had significantly higher mean E-DII scores compared to controls (*p* = 0.001).

Tertiles of E-DII, which ranged from −4.23 to + 3.89, were created based on the baseline E-DII. Tertile 1 represents subjects with lowest inflammatory potential of diet, Tertile 2 is intermediate and tertile 3 includes the participants with the highest inflammatory potential of diet.

Control characteristics across categories of E-DII are presented in Table 2. Participants in tertile 3 were significantly more likely to be diagnosed with diabetes (*p* = 0.001) and hypertension (*p* = 0.02). Subjects in tertile 3 consumed significantly more fried food (*p* = 0.006) and less boiled foods (*p* = 0.001). They also had significantly lower levels of physical activity (*p* < 0.001) and higher energy intake (*p* < 0.001) compare to subjects in tertile 1.

Odds ratios (ORs) and 95% confidence intervals (CIs) for the risk of CRC according to tertiles of E-DII are provided in Table 3. Results obtained from the multivariable adjusted continuous E-DII in relation to risk of CRC showed a significant positive (OR_continuous_ = 1.71, 95% CI: 1.41–2.07). When analysis was carried out with E-DII expressed as tertiles, subjects in the third (highest) tertile were five times more likely to have CRC compared to subjects in the first (lowest) tertile (OR_tertile 3 vs. 1_ = 5.08, 95% CI: 2.70–9.56; P-trend < 0.0001).

For the multivariable-adjusted model based on continuous of E-DII in relation to risk of CAP provided in Table 4. The results showed a significant positive association (OR_continuous_ = 1.30, 95%CI: 1.09–1.55). When analysis was performed using categorical E-DII (tertiles of E-DII) the highest tertile of E-DII versus to the lowest tertile showed significant positive association for the risk of CAP (OR_tertile 3 vs. 1_ = 2.33, 95% CI: 1.30–4.02; P-trend = 0.005).

## 4. Discussion

Our results show that adherence to a pro-inflammatory diet, represented by increased E-DII scores, increases the risk of both CRC and CAP. These associations remained significant even when analyses were adjusted for known CRC and CAP risk factors. Previous studies that mainly evaluated the association between DII scores and CRC have found similar results [10,11,19,26,42,43]. Our results not only confirmed the results of previous studies, but also have shown that the E-DII, independent of other risk factors, is associated with CAP and this is an important finding because adenomas are thought to be important pre-cancerous conditions. These findings add to the evidence supporting a causal relationship between increasing inflammatory potential of diet and risk of CRC.

Most previous studies have been conducted in Western countries. A recent meta-analysis reported that a healthy dietary pattern decreased the risk of CRC, while a ‘Western-style’ and ‘alcohol-consumption’ patterns increase the risk [44]. A recent systematic review of the published evidence on foods and beverages and colorectal cancer by the World Cancer Research Fund–American Institute for Cancer Research (WCRF-AICR) reported that there was evidence that processed meat and alcoholic drinks increase the risk of colorectal cancer. There also is evidence that red meat increases risk, while dairy products, wholegrains and foods containing dietary fibre reduce the risk of CRC and its precursors including CAP and a chronic state of colonic inflammation [45].

Finding a significant association between the E-DII and CRC/CAP in the Iranian population, with their unique dietary pattern, suggests a pivotal role for diet-associated inflammation in the development of CRC/CAP. Although the Iranian diet is in a transition from the traditional Iranian pattern to the Western pattern, this transition is not yet complete. Moreover, due to the age of our study population, study participants are likely to have consumed the traditional diet through their childhood, adolescence and early adulthood. Our traditional diet consists of high intake of carbohydrates and fried vegetables, relatively infrequent consumption of a variety of meat and oil (which varies according to socioeconomic status) and, rarely, alcohol [46]. Thus, our population can provide good contrasts for identifying associations between the dietary inflammation-associated intake and various health outcomes.

The DII score and, by logical extension, the E-DII score are based on multiple components, which include nutrients, food items and flavonoids, all of which have been shown to have an effect on inflammation [47,48,49]. The most important anti-inflammatory components are polyphenols and anti-oxidant foods, which have anti-inflammatory effect scores [26]. These anti-oxidant compounds can produce anti-inflammatory effects, especially locally through the action of local microbiota [50]. Antioxidants also can inhibit cancer initiation and progression through reduction in free oxidative agents such as reactive oxygen species (ROS) [51]. Phytochemical compounds of diet prevent colorectal cancer cell growth inhibition of the activation of NF-κB pathway [52], and inhibition of the cell cycle [53]. Another important component of the DII/E-DII score is dietary iron, which has pro-inflammatory properties. Because iron can produce free radicals in the body, it can act as an oxidant, inducing DNA damage in different cells, especially colonocytes because they have the most intimate contact with dietary iron. 

Processed and red meat are high in several inflammatory components such as proteins, total fat, trans fat, cholesterol, vitamin B12, saturated fatty acids and iron. Several studies have reported a positive association between processed and red meat consumption and risk of CRC [54]. Several factors including high iron, fat and protein intake and polycyclic aromatic hydrocarbons (PAHs) and heterocyclicamines (HCAs) have been suggested to explain this association [26,55,56,57]. High fat consumption can stimulate the secretion of secondary bile acids, which promote tumorigenesis [55,58]. Moreover, high amounts of free fatty acids in the colonic lumen may damage the integrity of colonocytes, which stimulates cellular proliferation leading to malignancy.

The DII score has the advantage of taking into account the totality of an individual’s diet so that the higher score indicates higher diet-related inflammatory capacity of the entire diet. The mechanism by which inflammation predisposes people to CRC has been explained previously [11,17,26]. It has been shown that systemic inflammation induces insulin resistance because inflammatory cytokines such as tumor necrosis factor-α (TNF-α) inhibits insulin receptors [59]. Insulin resistance can induce CRC and CAPs through the growth-promoting effects of elevated insulin, glucose, or triglycerides [10]. Moreover, activation of the cyclooxygenase-2 (COX-2) pathway may lead to focal proliferation, angiogenesis and mutagenesis [26]. COX-2 can be up-regulated by inflammatory cytokines such as interlukine-6 (IL-6), and down-regulated by anti-inflammatory dietary components of the DII.

This study has some limitations. Selection bias is inevitable in case-control studies; however, we recruited consecutive cases and controls from the same referral hospital to reduce this bias. Recall bias is another potential limitation inherent to the case-control design. Furthermore, we did not collect any information regarding family history of CAP of the participants.

Despite its weaknesses, this study has various strengths. Using a reliable and validated FFQ [60] helped us to reduce the measurement error. Another advantage is that we evaluated the association of E-DII with both CRC and CAP risk at the same time. Considering these exposures simultaneously increases confidence in inferring causality because CAP is a precursor condition for CRC. That these associations remained significant after adjusting for known risk factors of CRC instills yet more confidence in the result.

## 5. Conclusions

In conclusion, results from our study have shown that higher E-DII scores are associated with higher risk of CRC and CAP. Further prospective studies, especially feeding trials, are recommended to confirm our results.

## Figures and Tables

**Table 1:** The main characteristics of the participants, Iranian colorectal cancer (CRC) and colorectal adenomatous polyps (CAP) case-control study, Tehran, 2017–2018.

Variables	Controls (n = 240)	CRC ^€^ (n = 134)	CAP ^£^ (n = 130)	*p*-Value *	*p*-Value ^†^
Age (years) ^a,‡^	56 (50–61.75)	59 (49.25–64)	58 (51–64)	0.06	0.09
Gender (male) ^b,‡^	133 (55.4)	66 (51.2)	59 (45.4)	0.43	0.06
E-DII ^§ c^	−0.90 (1.37)	00.00 (1.26)	−0.41 (1.20)	**0.001**	**0.001**
BMI (kg/m^2^) ^a^	26.53 (24.10–29.40)	39.30 (37.17−40.90)	26.79 (23.86–29.40)	0.23	0.95
Smoking (yes) ^b^	42 (17.5)	26 (20.2)	27 (20.8)	0.53	0.11
Comorbidity(yes) ^b,¥^	41 (17.1)	21 (16.3)	40 (30.8)	0.84	**0.002**
Diabetes(yes) ^b^	19 (7)	11 (10)	20 (30)	**0.001**	**0.03**
hypertension(yes) ^b^	12 (8.3)	6 (4.3)	12 (39)	0.07	**0.01**
coronary heart disease(yes) ^b^	10 (1.8)	4 (2)	8 (19)	0.009	**0.008**
Family history of cancer in first degree relatives (yes) ^b^	89 (32.9)	66 (51.2)	48 (36.9)	**0.001**	0.43
Family history of colorectal cancer in first degree relatives (yes) ^b^	18 (7.5)	10 (7.8)	17 (13.1)	0.15	0.08
Common ways of cooking food ^b^					
Fried	55 (22.9)	40 (31)	18 (13.8)	**0.001**	0.06
Boiled	81 (33.8)	41 (31.8)	34 (26.2)	0.75	0.82
Grilled	5 (2.1)	0 (0)	4 (3.1)	**0.02**	0.52
Steam cook	3 (1.3)	2 (1.6)	0 (0)	0.32	**0.006**
Combined	96 (40)	46 (35.7)	74 (56.9)	**0.001**	0.06
Level of salt intake ^b^					
Low	127 (52.9)	44 (34.1)	44 (34.1)	**0.002**	**0.002**
Normal	79 (32.9)	64 (49.6)	64 (49.6)	0.08	0.08
High	34 (14.2)	21 (16.3)	21 (16.3)	0.07	0.07
Physical activity (MET ^Ψ^/h/day) ^a^	39.02 (36.51–41.33)	39.30 (37.17–40.90)	37.75(34.67−40.40)	0.85	**0.003**
Monthly intake of 50,000 IU Vitamin D supplement (yes) ^b^	56 (23.3)	28 (21.7)	40 (30.8)	0.72	0.11
Daily intake of 500 mg Calcium supplement (yes) ^b^	35 (14.6)	28 (21.7)	32 (24.6)	0.08	**0.01**
Energy intake (kcal/day) ^a^	2282.28	2188.08	2204.02	0.35	0.35
	(1941.24−2662.76)	(1843.91−2649.87)	(1827.92−2774.53)		

^€^ CRC = Colorectal cancer. ^£^ CAP = Colorectal adenomatous polyp. ^§^ Energy-adjusted DII. ^Ψ^ Metabolic Equivalent of Tasks ^a^ Mean (range). ^b^ Number (Percent). ^c^ Mean (SD). ^‡^ Matched variables of the study. * *p*-value between CRC and controls, ^†^ *p*-value between CAP and controls. ¥ comorbidities are defined as diabetes, hypertension and coronary heart disease. MET: Metabolic equivalent/independent sample t-test and Mann–Whitney were used for continuous variables with normal distribution and non-normal distribution respectively and Chi-square was used for categorical variables.

**Table 2:** Participant characteristics by tertile of the energy-adjusted dietary inflammatory index score (E-DII) among controls, Iranian CRC and CAP case-control study, Tehran, 2017–2018.

	Tertile 1	Tertile 2	Tertile 3	*p*-Value ^¥^
	<−1.13	−1.13−0.03	>0.04	
Age (years) ^a^	57.76 (8.30)	53.21 (9.87)	52.76 (9.74)	0.06
Gender (male) ^b^	58 (55.8)	47 (58)	28 (50.9)	0.71
Body mass index (BMI) (kg/m^2^) ^a^	27.06 (4.12)	26.56 (3.69)	27.23 (4.22)	0.07
Current smoking(yes) ^b^	17 (16.3)	16 (19.8)	9 (16.4)	0.8
Comorbidity (yes) ^†,b^	5 (9.1)	13 (16.0)	23 (22.1)	0.11
Diabetes(yes) ^b^	2 (3.2)	6 (8)	12 (11)	**0.001**
Hypertension(yes) ^b^	2 (3.4)	4 (5)	8 (9)	**0.02**
Coronary heart disease(yes) ^b^	1 (2.5.)	3 (2)	3 (2.1)	0.6
Family history of cancer in first degree(yes) ^b^	38 (36.5)	23 (28.4)	18 (32.7)	0.5
Colorectal cancer family history in first-degree relative(yes) ^b^	7 (6.7)	6 (7.4)	5 (9.1)	0.86
Common ways of cooking food ^b^				
Fried	9 (17.3)	20 (24.7)	26 (30.9)	0.006
Boiled	45 (43.3)	19 (23.5)	17 (30.9)	0.001
Grilled	2 (1.9)	3 (3.7)	0 (0)	0.2
Steam cook	1 (1.0)	1 (1.2)	1 (1.8)	0.69
Combined	38 (36.5)	38 (46.9)	20 (36.4)	0.07
Physical activity (MET^Ψ^/h/day) ^a^	41.33 (13.04)	39.02 (7.28)	39.20 (4.77)	**<0.001**
Monthly intake of 50,000 IU Vitamin D supplement (yes) ^b^	26 (25.0)	15 (18.5)	15 (27.3)	0.43
Daily intake of 500 mg Calcium supplement (yes) ^b^	14 (13.5)	10 (12.3)	11 (20.0)	0.42
Energy intake (kcal) ^a^	2174 (510)	2336 (606)	2778 (847)	**<0.001**

^a^ Mean (SD). ^b^ Number (percent) ¥ p-value represent the difference between tertile 3 vs. tertile 1 of DII. † Comorbidities are defined as diabetes, hypertension and coronary heart disease. ^Ψ^ Metabolic Equivalent of Tasks

**Table 3:** Odds ratios and 95% confidence intervals for the association between energy-adjusted DII (E-DII) and CRC, Iranian CRC and CAP case-control Study, Tehran, 2017–2018.

E-DII	Tertiles of E-DII					P-Trend	Continuous OR	95% CI
	I	II		III			−4.23 to 3.89	
	<−1.13	−1.13–0.03		>0.04				
N: control/CRC	79/24	80/42		81/63				
		OR	95% CI	OR	95% CI			
Model 1	1.00 (ref)	2.43	1.339−4.43	5.48	3.03−9.92	.0001	1.67	1.40−2.00
Model 2	1.00 (ref)	2.64	1.40−4.99	5.08	2.70−9.56	.0001	1.71	1.41−2.07

Model 1. Adjusted for age and sex. Model 2. Adjusted for Age, Sex, Physical Activity, Level of salt intake, Diabetes, Hypertension, Coronary heart disease, Smoking, Family history of cancer in first degree, Common ways of cooking food and calcium supplement intake.

**Table 4:** Odds ratios and 95% confidence intervals for the association between Energy-adjusted DII (E-DII) and CAP, Iranian CRC and CAP Case-Control Study, Tehran, 2017–2018.

E-DII	Tertiles of E-DII					P-Trend	Continuous OR	95% CI
	I	II		III			−4.23–3.89	
	<−1.13	−1.13–0.03		>0.04				
N: control/polyp	79/39	80/43		81/48				
		OR	95% CI	OR	95% CI			
Model 1	1.00 (ref)	1.54	0.90−2.63	2.39	1.39 −4.11	.002	1.33	1.13−1.58
Model 2	1.00 (ref)	1.46	0.82−2.58	2.33	1.30−4.02	.005	1.30	1.09−1.55

Model 1. Adjusted for age and sex. Model 2. Adjusted for Age, Sex, Physical Activity, Level of the salt intake, Diabetes, Hypertension, Coronary heart disease, Smoking, Family history of cancer in first degree, common ways of cooking food and calcium supplement intake.

## Supplementary Materials

The following are available online at https://www.mdpi.com/2072-6643/11/6/1213/s1, Figure S1: Food Frequency Questionnaire.
