# Supplementary material for: Dietary Inflammatory Index and Odds of Colorectal Cancer and Colorectal Adenomatous Polyps in a Case-Control Study from Iran
Source: Nutrients. 2019 May 28;11(6):1213. doi: 10.3390/nu11061213 (PMC6628332; doi:10.3390/nu11061213)
Supplement: Supplementary file 1 [file nutrients-11-01213-s001.pdf]

In the Name of God
Research Institute of Endocrinology and Metabolism
Shaheed Beheshti University of Medical Sciences
Food Frequency Questionnaire (FFQ) "Tehran Lipid and Glucose Study"

Name: Acceptance Code: Age:

No.	DAIRY FOODS	HOW MUCH	HOW OFTEN				CONSIDERATIONS
			per day	per week	per month	per year	
1	Lavash bread (refined grains)	1 loaf					
2	Barbari bread (refined grains)	1 loaf					
3	Sangak bread (whole grains)	1 loaf					
4	Taftoon bread (refined grains)	1 loaf					
5	Baguette bread	1 loaf					
6	Cooked rice	1 dinner plate					normal: full:
7	Cooked pasta	1 spatula					Flat head: full:
8	Potato	1 medium					
9	French fries	1 slice					
10	Baked vermicelli (soup noodle)	1 cup					
11	Ash noodle	1 cup					
12	Cookies (stating the type)	1 number					type:
13	Crackers (crispy biscuits)	1 number					
14	Types of cakes	1 medium slice					(Including homemade cakes, cookies, T-tops, etc.)
15	Corn	1 medium					
16	Cooked barley or bulgur	1 cup					
17	Lentil	1 cup					
18	Beans	1 cup					
19	Pea	1 cup					
20	Baked bean	1 cup					
21	Soy bean	1 cup					
22	Mung	1 cup					
23	Cotyledon	1 cup					
24	Beef or calf	1 slice of stewed					
25	lamb meat	1 slice of stewed					
26	Ground beef	1 tablespoon					
27	Hen and Chickens	1 medium piece					Drumstick: chest:

							wings: with peel: peeled:
28	Fish (except tuna) stating the type	1 medium piece (one palm full)					The amount of: type:
29	Tuna (canned)	1/2 cans					Is it discarded oil? Yes * No *
30	Heart, liver and kidney	1 skewer					
31	Hamburger	1 number					
32	kielbasa	1 Cut					
33	Sausage	1 number (Germany * cocktails *)					
34	Egg	1 Number					
35	Tripe and Rennet	1 piece					
36	Tongue	1 whole number					
37	Brain	1 whole number					
38	Head	1 palm					
39	Leg	1 Number					If certain amount and components is used, noted.
40	Pizza	1 number					
41	Low-fat milk (less than 2%)	1 cup					
42	Whole milk (greater or equal to 2%)	1 cup					
43	Chocolate milk	1 cup					
44	Ooze Yogurt	1 tablespoon					
45	Regular yogurt	1 Bowl/cup					
46	Full fat yogurt	1 Bowl/cup					
47	Cheese	1 pat					
48	Cream cheese	1 pat					
49	Dough	1 cup					
50	Cream	1 tablespoon					
51	Traditional ice cream	Half cup					how months of year?
52	Non-traditional ice cream	1 number					how months of year?
53	Butter	1 pat					
54	Margarine	1 pat					
55	Dried whey	1 tablespoon					
56	Shredded lettuce	1 cup					
57	Tomato	1 medium					
58	Cucumber	1 medium					
59	Fresh Herbs	1 small plate					
60	Cooked vegetables	1 cup					

	(soup, rice, etc.)						
61	Pumpkin	1 medium					
62	Stewed pumpkin	1 medium					
63	Baked Eggplant	1 medium					
64	Boiled Celery	1 cup					
65	Green peas cooked	1 cup					
66	Green beans cooked	1 cup					
67	Raw carrots	1 medium					
68	Cooked carrots	1 medium					
69	Garlic	1 clove					
70	Raw onion	1 small					
71	Fried onions	1 tablespoon					
72	Cabbage varieties	1 Bowl/cup					
73	Bell peppers	1 medium					
74	Cooked spinach	1 cup					
75	Turnip	1 medium					
76	Small green pepper	1 medium					
77	ketchup	1 tablespoon					
78	Pickles	1 cup					how months of year?
79	SHOOR	1 cup					how months of year?
80	Pickled cucumber	1 medium					
81	Cantaloupe	1/4 number					
82	Melon	1 medium slice					
83	Watermelon	1 medium slice					
84	Pear	1 medium					
85	Apricot	1 medium					
86	Cherries	1 small plate					
87	Apple	1 medium					
88	Peach	1 medium					
89	Nectarines	1 medium					
90	Prunus	1 medium					
91	Fresh figs	1 medium					
92	Dried figs	1 medium					
93	Grape	1 medium bunch					
94	Kiwi	1 medium					
95	Grapefruit	1 medium					
96	Orange	1 medium					
97	Persimmon	1 medium					
98	Tangerine	1 medium					
99	Pomegranate	1 medium					
100	Date	1 medium					
101	Plums (yellow and red)	1 medium					
102	Strawberry	1 number					

103	Banana	1 medium					
104	Sweet lemon	1 medium					
105	Lemon	1 medium					
106	Orange juice	1 cup					
107	Apple juice	1 cup					
108	Cantaloupe juice	1 cup					
109	Raisins	1 tablespoon					
110	Fresh berries	1 small plate					
111	Dried berries	1 number					
112	Dried fruits	1 number					
113	Green Olive	1 number					
114	Canned fruits	1 can					
115	Packaged fruit juices	1 number					
116	Solid vegetables oils	1 tablespoon					
117	Oil	1 tablespoon					
118	Olive oil	1 tablespoon					
119	Ghee	1 tablespoon					
120	Mayonnaise	1 tablespoon					
121	Peanut	1 number					
122	Almond	1 number					
123	Walnut	1whole number					
124	Pistachios	1 number					
125	Hazelnut	1 number					
126	Seeds (watermelon, pumpkin, sunflower)	1 Bowl/cup					
127	Sugar cube, comfit	1 number					
128	Sugar	1 teaspoonful					
129	Honey	1 teaspoonful					
130	Jams (by type)	1 tablespoon					
131	Industrial Or cola beverages.	1 cup					
132	Dried sweets	1 medium					
133	Cream Sweets	1 medium					
134	GAZ	1 medium					
135	SOHAN	1 Piece					
136	Puff	1 pack					
137	Chocolate	1 number					
138	Tea	1 cup					
139	Salt	1 tsp					Type:
140	Chips	1 pack					
141	Coffee	1 cup					
142	Lemon juice	1 teaspoonful					
143	Candy	1 medium					
144	Baked mushrooms	Half cup					
145	Homemade HALVA	1 tablespoon					

146	Sesame pudding	1 tablespoon					
147	Spices	1 tsp					

The date of delivery of food questionnaires to residents:

Name of dietitian responsible for completing FFQ:

delivery date to Nutrition expert:
